# Developing a Novel Pediatric Eye Chart Assessing Visual Acuity by Minimum Separable Threshold

**DOI:** 10.3390/children11040397

**Published:** 2024-03-27

**Authors:** Yo Iwata

**Affiliations:** Department of Rehabilitation, Orthoptics and Visual Science Course, School of Allied Health Sciences, Kitasato University, 1-15-1 Kitasato, Sagamihara 252-0373, Japan; iwatayo@kitasato-u.ac.jp; Tel.: +81-42-778-9671; Fax: +81-42-778-9684

**Keywords:** Landolt ring, amblyopia, minimum separable threshold, pediatric ophthalmology, visual acuity test

## Abstract

The purpose of this study was to develop a new pediatric acuity chart that can assess the minimum separation threshold by incorporating the minimum separation threshold into the picture. To overcome the design limitations of the Landolt ring, two designs of highly versatile minimum separable thresholds that can be easily incorporated into a picture were created: a black, filled circle (the “Circle”) and a segment (the “Square”), both with the same break as in the Landolt ring. The three designs—the Landolt ring, Circle, and Square—were used to evaluate and compare the differences in the visual acuity of 21 healthy adults. No significant differences were observed between the results of the visual acuity tested with the Landolt ring, Circle, and Square (Landolt ring vs. Circle: *p* = 0.92, Landolt ring vs. Square: *p* = 0.31, Circle vs. Square: *p* = 0.40). The Bland–Altman analysis revealed no fixed errors between the Landolt ring and Circle and between the Landolt ring and Square (95% CI: −0.09–0.08, −0.09–0.12). Proportional errors were also not observed (*p* = 0.68, *p* = 0.41). The Landolt ring, Circle, and Square designs obtained equal results in visual acuity, thus achieving the successful development of a novel pediatric visual acuity chart using these designs.

## 1. Introduction

Amblyopia is a neurodevelopmental disorder of the visual cortex that results from abnormal visual experiences in early life [1] in which good visual acuity cannot be achieved even with refractive correction [2]. Regarding the prevalence of amblyopia, Wang et al. in 2011 reported a prevalence of 3% [3], and in 2018–2019, a meta-analysis reported a prevalence of 1.75% [4] by Hashemi et al. and 1.44% [5] by Fu et al. There are differences in prevalence by ethnicity, with a lower prevalence in individuals of African descent and a higher prevalence in those of European descent [6]. In 2019, there will be 99.2 million amblyopia patients worldwide; however, projections predict that by 2030, this number will increase to 175.2 million and by 2040 to 221.9 million [5]. There is no gender difference in the prevalence, and unilateral amblyopia is more common than bilateral amblyopia, with anisometropic amblyopia being the most common among all types of amblyopia [3,6]. According to a report that investigated the psychological effects of amblyopia on patients, an assessment using the Hopkins symptom checklist, which quantitatively analyzes psychosomatic, anxiety, obsessive compulsive, interpersonal irritability, and depressive symptoms, found that all indicators were significantly higher in the amblyopia group than in the control group [7]. Furthermore, in a report investigating the functional limitations of amblyopic patients, 95% of all patients were aware of some functional limitations [8]. It is also reported that the estimated lifetime risk of bilateral visual impairment is nearly doubled by the presence of amblyopia [9]. Treatment of amblyopia includes full refractive glasses [10], eye-patch with occlusion [11], dichoptic treatment [12], perceptual learning [13], and action gameplay [14]. More effective methods of treating amblyopia have been discussed, but the most effective methods are still unknown, and all methods require a long period of treatment. While there is no age limit for amblyopia treatment, there are certain ages at which treatment is more effective [15,16,17,18]. Reportedly, treating amblyopia earlier results in better outcomes, and the peak of treatment efficiency is before the age of 3 years [19]. In a study of amblyopia treatment using an occlusion dose monitor that allows complete control of occlusion time, the amblyopia treatment time required to achieve a visual acuity gain of 0.2 logMAR for 3–8-year-olds was 170 h for 4-year-olds compared to 236 h for 6-year-olds [16]. For 5.4–15.8 year-olds, 220 h were reported to be needed at age 6, 490 h at age 8.4, and 426 h at age 12.9 [20]. A meta-analysis by the Pediatric Eye Disease Investigator Group, which classified patients with moderate-to-severe amblyopia into 3–5 years, 5–7 years, and 7–13 years of age, also showed that the effect of treatment for amblyopia with occlusion therapy was significantly lower in the 7–13 age group, and the effect was highest in the 3–5 age group when limited-to-severe cases were included [21]. From these reports, it is clear that visual sensitivity peaks in early childhood and then gradually weakens, and a delay in the start of treatment would significantly increase the duration of treatment. In other cases, good visual acuity cannot be achieved by the treatment [16]. Thus, early detection and treatment for amblyopia are essential.

In Japan, the visual acuity tests conducted at the 3-year-old health screening and health assessment at entry to elementary schools are opportunities for early detection of amblyopia [22]; despite some differences, many countries follow a similar schedule to screen for amblyopia [23,24,25,26]. The Landolt ring is generally used in the visual acuity tests in these screening programs. However, young children often do not understand the point of the visual acuity test using the Landolt ring. To evaluate visual acuity using a Landolt ring, the patient must be asked to point in the direction of the gap, up, down, left, or right, or by holding a model similar to a Landolt ring, have the patient match the slit to the distant Landolt ring. Indeed, the visual acuity examination using the Landolt ring is administered correctly in 70% of 3-year-olds [27] and only in as few as 48% of children aged 23–70 months (median: 42.5 months) [28]. A Japanese study revealed that 81% of patients in the hospital with amblyopia were undiagnosed when tested with Landolt ring at the 3-year-old health screening, but were diagnosed with amblyopia when tested with Landolt ring at a health assessment made in entry to elementary schools, potentially due to the test’s complexity for young children to understand [29]. Recently, photoscreeners have been widely adopted as screening devices for amblyopia. Photoscreener is a compact device that can easily and quickly detect risk factors for amblyopia, such as refractive error and strabismus, by having the patient fixate on a glowing optotype for a few seconds [30]. However, since this device only evaluates the refractive value and eye position, it is essential to make a direct evaluation of visual acuity in order to diagnose, evaluate the treatment, and monitor the course of amblyopia. It has been reported that not all children have amblyopia, even when they have moderate or high hyperopia [31,32].

**Figure 1:**
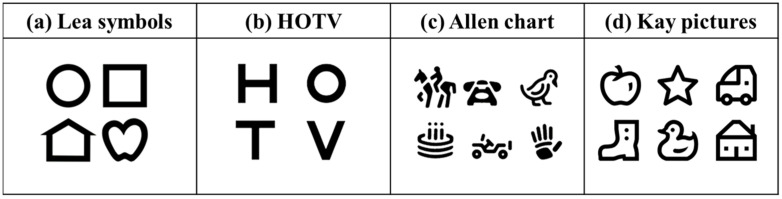
Displays the internationally used pediatric visual acuity chart. (**a**) Lea symbols chart. (**b**) HOTV. (**c**) Allen chart. (**d**) Kay pictures.

Children who cannot understand the visual acuity test with the Landolt ring and those with poor visual acuity are recommended to visit an ophthalmology clinic. Ophthalmology clinics typically use the Lea symbol chart (Figure 1a), HOTV (Figure 1b), Allen chart (Figure 1c), and Kay picture (Figure 1d) as visual acuity tests for children. Lea symbols consist of geometric shapes such as circles, squares, hearts, and houses; HOTV consists of four simple letters: H, O, T, and V; Allen charts consist of shapes familiar to children, such as horse, telephone, bird, cake, car, and hand; and Kay pictures consist of shapes for children, such as apple, star, car, shoe, duck, and house. However, the International Organization for Standardization (ISO) recommends the evaluation of the minimum separable threshold for the test of visual acuity [33]; in contrast, the visual charts mentioned above are not evaluated by the minimum separable threshold. Furthermore, the results obtained through these visual acuity charts do not correspond to those obtained through tests using other charts, such as with the Landolt ring or the Early Treatment Diabetic Retinopathy Study (ETDRS) chart, as visual acuity assessed with Lea symbols comes out higher than that assessed by ETDRS [34] and the Landolt ring [35,36]. Visual acuity measured with the HOTV comes out higher than that evaluated by the Landolt ring, ETDRS chart, or Lea symbols [37,38,39]. The visual acuity assessed by the Allen chart also turns out higher than that assessed by the Landolt ring [37]. Kay pictures evaluate visual acuity higher than the ETDRS chart [40].

Although numerous pediatric visual acuity charts exist, none are evaluated by the minimum separable threshold as recommended by the ISO. Therefore, this study aimed to develop a pediatric visual acuity chart that assesses visual acuity by evaluating the minimum separable threshold. 

**Figure 2:**
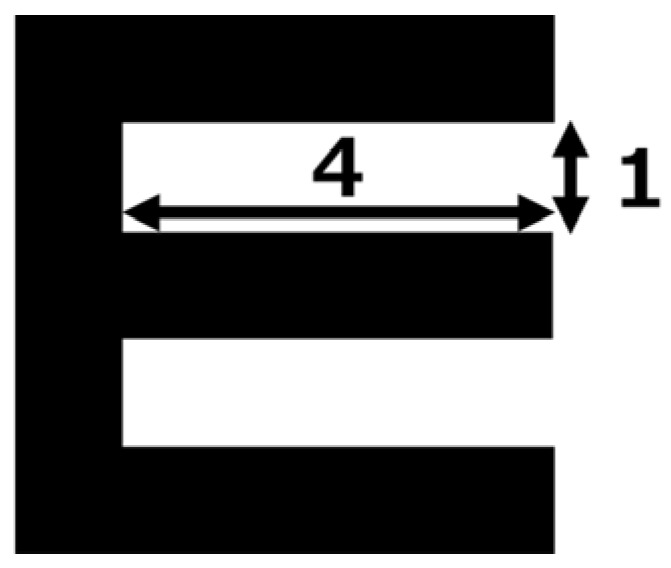
The width of the break in the E chart optotype is four times its length.

**Figure 3:**
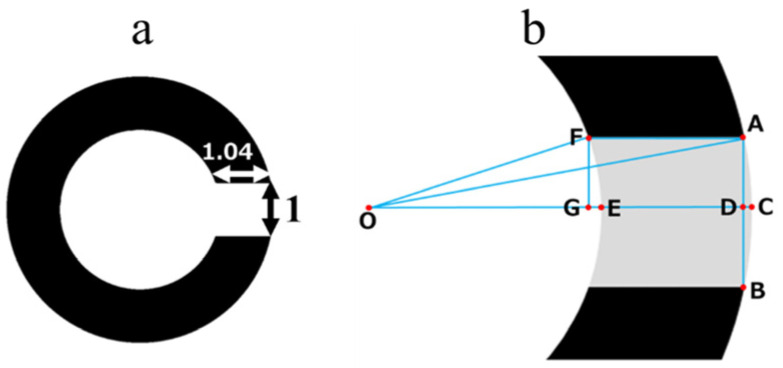
(**a**) The width of the break in the Landolt ring is 1.04 times its length. (**b**) Details of calculation method.

“Minimum separable threshold” is defined as “the smallest visual angle formed by the eye and two separate objects at which a patient can discriminate them individually [41]. The Landolt ring and E chart are two types of visual acuity tests that evaluate minimum separable threshold. However, although the lengths of the breaks in the two types are the same, their widths are not. The width of the break in the E chart optotype is four times its length (Figure 2); whereas, in the Landolt ring, it is only approximately 1.04 times its length (Figure 3a). The calculation method is shown in Figure 3b. 

We assumed the length of the break of the Landolt ring to be 1, the diameter of the outer circle as 5, and its radius OA as 2.5. The length of AD corresponds to half the length of the break and, therefore, equates to 0.5. Per the Pythagorean theorem, AD^2^ + OD^2^ = OA^2^, therefore, 0.5^2^ + OD^2^ = 2.5^2^. Accordingly, OD = √6. The length of the radius and OE of the inner circle of the Landolt ring is 1.5. Therefore, DE = OD − OE = √6 − 1.5. The length of OE corresponds to the radius of the inner circle of the Landolt ring and, therefore, equates to 1.5. By the Pythagorean theorem, FG^2^ + OG^2^ = OF^2^ = 0.5^2^ + OG^2^ = 1.5^2^; therefore, OG = √2 and GE = OE − OG = 1.5 − √2, and AF = DE + GE = √6 − 1.5 + 1.5 − √2 ≈ 1.04. 

Therefore, the E chart reportedly estimates visual acuity better than the Landolt ring [42,43,44]. This study aims to create an optotype with results that correspond to the Landolt ring to create a pediatric visual acuity chart that evaluates the minimum separable threshold resulting in a visual acuity equivalent to that assessed by the Landolt ring, an optotype consisting of a Landolt ring with a break that is 1.04 times as wide as its length is required.

**Figure 4:**
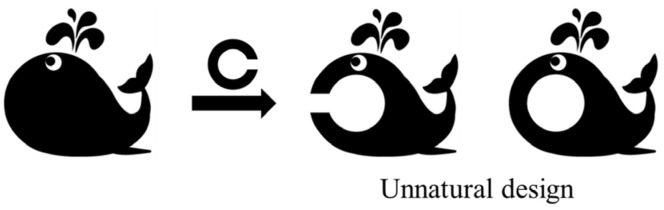
Incorporating the Landolt ring directly into the picture creates an unnatural esthetic, and the design variations also become very limited.

**Figure 5:**
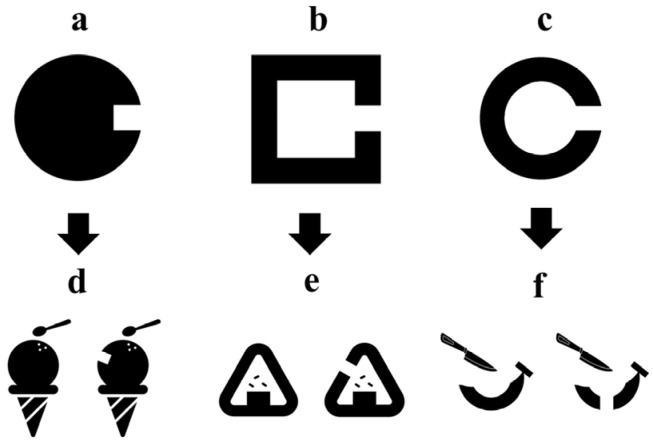
(**a**) The minimum separable threshold design with a black circle with a break. (**b**) The minimum separable threshold design comprising a segment with a break. (**c**) Landolt ring. (**d**–**f**) Pediatric visual acuity chart designs, each incorporating optotypes (**a**–**c**).

In developing the pediatric visual acuity chart that evaluates the minimum separable threshold, we first considered using a two-image method. One of the two drawings would be shown with a minimum separable threshold, and the patient would answer which drawing has the break. However, the Landolt ring has limited versatility when inserted in drawings as it results in unnatural designs (Figure 4), thus restricting the variations of optotypes. This led us to devise novel designs that measure the minimum separable threshold: a “Circle”, which consists of a break in a black circle (Figure 5a), and a “Square”, which consists of a segment with a break (Figure 5b). These designs will serve as a foundational structure for a picture optotype with a natural esthetic, incorporating the minimum separable threshold. For example, the Circle design can be incorporated into a picture like the ice cream shown in Figure 5d, the Square design can be incorporated into a picture like the rice ball shown in Figure 5e, and the Landolt ring design can be incorporated into a picture like the banana shown in Figure 5f. However, it is unknown whether these designs can evaluate visual acuity to the same level as the Landolt ring, and this will be investigated in this study.

## 2. Methods

### 2.1. Participants and Methods

Individuals aged 18–39 years old (mean ± standard deviation, 23.2 ± 5.1) with uncorrected visual acuity of logMAR values was better than 1.0 and less than −0.1 were included. Those with astigmatism of ≥−1.50 diopter (D) as measured by an autorefractometer (ARK-1S, NIDEK, San Jose, CA, USA) or ophthalmological diseases other than refractive errors were excluded. Visual acuity was measured using the standard ETDRS method (ETDRS Visual Acuity Chart, T.M.I. Company, New Castle, DE, USA), and refractive values were measured in a non-cycloplegic autorefractor, both of which were performed prior to testing. Finally, 21 right eyes of 21 participants were analyzed.

The Landolt ring (Figure 5c), black circle with break (“Circle”, Figure 5a), and segment with break (“Square”, Figure 5b) were used to test visual acuity (Supplementary Materials). In all optotypes, the design was adjusted to the standardized ratio such that the width of the break was 1.04 times its length. A 12.9-inch monitor (iPad Pro/Apple, Cupertino, CA, USA, 2048 × 2732, 264 pixels per inch) was used to display the visual acuity. The test was conducted at a 5 m distance. The standard method of assessment designated by the ETDRS chart [45] was followed to evaluate uncorrected visual acuity. The total visual acuity value was calculated as the sum of the participant’s highest visual acuity value (in which all answers were correct) to the number of items they answered correctly in the next row (×0.02). The optotype was represented by a single letter. The Landolt ring, Circle, and Square were presented in a random order. The room illuminance was set at 600–800 lx, and the illuminance of the visual acuity chart was set at 475–625 rad lux. Illuminance was measured by Luxmeter LX-01 (Shimadzu scientific instrument, Columbia, MD, USA) once on each examination day. The optotype used for the visual acuity chart was completely black and the background was completely white, with a contrast ratio of at least 85%.

### 2.2. Ethical Consideration

This study complies with the Declaration of Helsinki and was approved by the Kitasato University Faculty of Health Sciences Ethics Review Committee (ethics review committee No. 11000465, approval No. 2022-021). The Ethics Committee approval date is 27 September 2022. All procedures conformed to approved guidelines. Informed consent was obtained from all participants after explaining to them the nature of the study and its possible consequences.

### 2.3. Statistical Analysis

The visual acuity values between the three designs were compared using the Wilcoxon signed-rank sum test and corrected by the Bonferroni method [46,47]. We also analyzed the fixed error and proportional error of the Circle and Square compared with the Landolt ring using the Bland–Altman plot and Spearman’s rank correlation coefficient [48]. A Shapiro–Wilk test was performed to analyze the sample distribution [49]. A significance level of <5% indicated a statistically significant difference. Only when the Bonferroni method was used, the significance level was determined as *p* < 0.017. The statistical software used was BellCurve for Excel Version 4.06 (Social Survey Research Information, Tokyo, Japan).

## 3. Results

**Figure 6:**
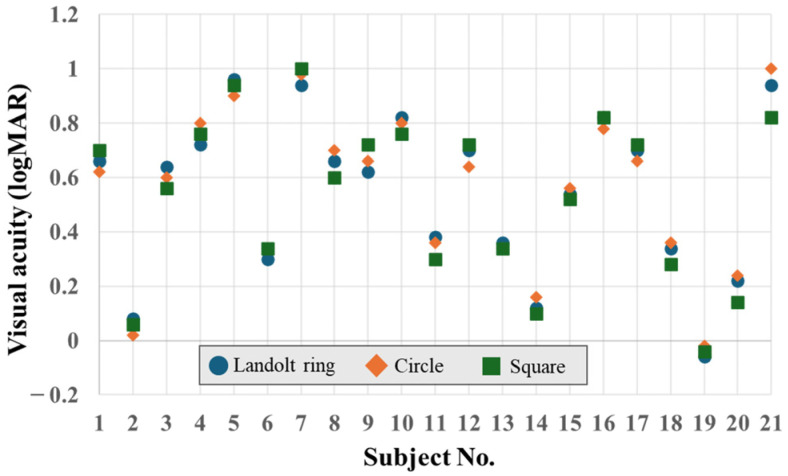
The visual acuity, as measured by the Landolt ring, Circle, and Square in the individual participants, is also shown. The largest difference in visual acuity was in subject no. 21 of 0.18.

**Figure 7:**
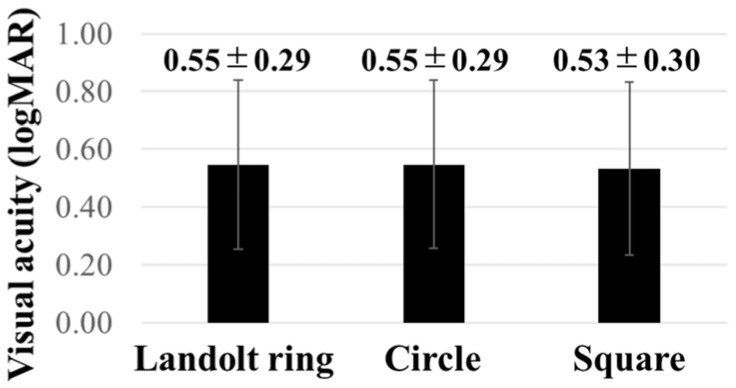
The visual acuity assessed by the Landolt ring, Circle, and Square are presented. No significant difference was observed between the three groups.

The visual acuity results of the test with the Landolt ring, Circle, and Square in the individual participants are shown in Figure 6. The results of the Shapiro–Wilk test showed *p* values of 0.39, 0.40, and 0.19 for the Landolt ring, Circle, and Square, respectively. The largest difference in visual acuity was 0.18 for subject No. 21, which was observed between the Landolt ring and the Square. The mean visual acuities with the Landolt ring, Circle, and Square were 0.55 ± 0.29, 0.55 ± 0.29, and 0.53 ± 0.30, respectively (Figure 7); no significant difference was observed between all three (Landolt ring vs. Circle; *p* = 0.92, Landolt ring vs. Square; *p* = 0.31, Circle vs. Square; *p* = 0.40, Table 1). The median (Q2) and interquartile ranges (Q3–Q1) for Landolt ring, Circle, and Square were 0.64 and 0.38, 0.62 and 0.44, and 0.60 and 0.46, respectively (Table 2).

**Figure 8:**
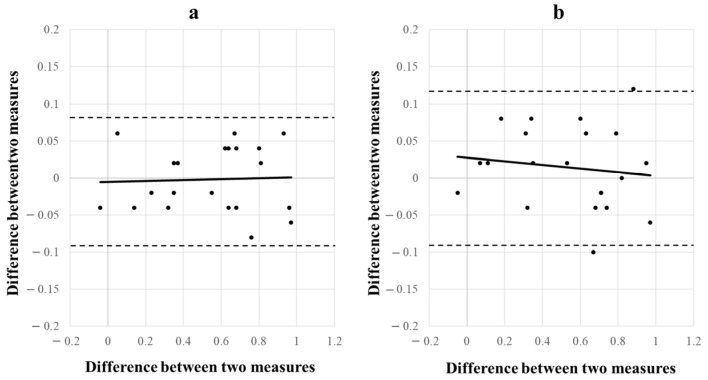
(**a**) Bland–Altman plot of the Landolt ring and Circle. (**b**) Bland–Altman plot of the Landolt ring and Square. Neither had fixed or proportional errors.

The Bland–Altman plot comparing the Landolt ring and Circle and the Landolt ring and Square is shown in Figure 8. No significant difference was observed between the Landolt ring and Circle in terms of fixed or proportional error (95% CI: −0.09–0.08; *p* = 0.68, Figure 8a). No significant difference was observed between the Landolt ring and Square in terms of fixed or proportional error (95% CI: −0.09–0.12; *p* = 0.41, Figure 8b).

## 4. Discussion

This study showed that visual acuity results equivalent to the Landolt ring could be obtained with the Circle and Square that assess the minimum separable threshold. This is attributed to the definition of the minimum separable threshold and also to the fact that the ratio of the length and width of the break were standardized to 1:1.04.

**Figure 9:**
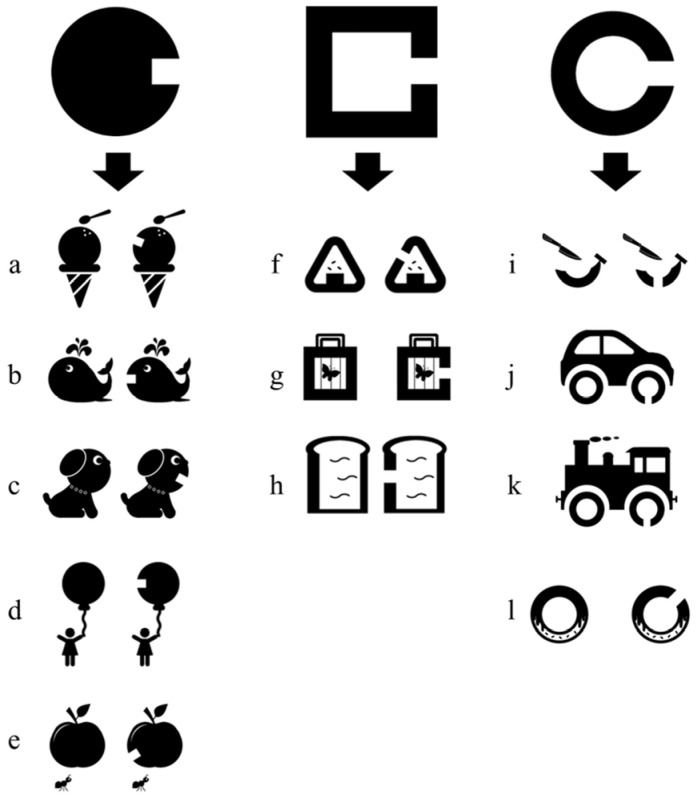
Picture optotypes applying the Landolt ring, Circle, and Square designs. Visual acuity is evaluated by asking the patient which of the two images has a break. Theoretically, all optotypes (the Landolt ring, Circle, and Square) should achieve equivalent results of visual acuity. (**a**) Ice cream; (**b**) Whale; (**c**) Dog; (**d**) Balloon; (**e**) Apple; (**f**) Rice ball; (**g**) Insect cage; (**h**) Bread; (**i**) Banana; (**j**) Car; (**k**) Train; (**l**) Doughnut.

These results show that theoretically, the optotypes applying the Landolt ring, circle and square should render equivalent results (Figure 9). This pediatric optotype evaluating the minimum separable threshold is obtained by choosing the correct answer from two options. For example, Figure 9c is accompanied by the question asking the patient, “Which dog’s mouth is open?” Figure 9i by “Which banana is cut?” and Figure 9j by “Which tire is broken?” to test visual acuity. Since it has been reported that children typically master the use of “left” and “right” only after the age of 7 [50], it is advisable to have them point rather than verbalize directions when using a visual acuity chart. While children can point in a direction by age 2 [51], the examiner might struggle to discern the optotype to which the child is indicating. Thus, we suggest that a more effective method could be to give the child a picture with a hole (e.g., in Figure 9, the picture of a cut banana) and instruct them to move it left or right, making it easier to determine their response. This study revealed that the minimum separable thresholds assessed by the circle and square provide the same results as the Landolt ring, thus suggesting that these designs applied in optotypes for children should provide equivalent parameters of visual acuity. Therefore, for example, using the optotype in Figure 9c at first and switching to the optotype in Figure 9i midway through when the child becomes bored may provide an opportunity for the child to maintain his/her concentration and continue to perform the vision test. Further research should be conducted on this topic. In addition, the Landolt ring is superior in detecting with-the-rule astigmatism and against-the-rule astigmatism because it has four directions (up, down, left, right) [52]. On the other hand, the pediatric optotype developed in this study is not capable of selectively displaying the four directions and is, therefore, considered inferior to the Landolt ring in detecting astigmatism.

Pediatric visual acuity charts used internationally have a host of problems; for example, their results do not correspond with the Landolt ring and ETDRS charts [34,35,36,37,38,39,40]; when they are based on a drawing, the visual acuity assessment varies between optotypes [40]; they lack variation in drawing types, and lastly, they do not evaluate minimum separable threshold as recommended internationally [33]. The novel pediatric visual acuity chart that applies the minimum separable threshold adopted in this research is superior to and provides solutions to the shortcomings of existing pediatric visual acuity charts.

Although this study is about a pediatric visual acuity chart, adults were included in the study, and the sample size is small. Adults were chosen as the subjects because it was felt that by including adults, the visual acuity chart evaluations in this study could be analyzed more accurately compared to those in children. Contrarily, it is still unclear how useful and effective these visual acuity charts will be in the pediatric population; hence, studies with children are essential. 

Early assessment of visual acuity is essential for early detection and treatment of amblyopia. However, only 56% of 31–36 month olds cooperated in the visual acuity examination using a Lea symbols chart [28], 92% among kids younger than six years [53], 75% among children aged 3–3.5 years [39], and 71% of 3–3.5-year-olds cooperated among kids taking the HOTV test [39], which shows that lack of cooperation among kids around the age of 3 years is often encountered. Considering that the earlier amblyopia treatment is initiated, the better the course of amblyopia, these success rates are not high enough, and an even higher success rate is required for visual acuity testing. Therefore, the success rate of visual examinations for children using the eye chart developed in this study should be further investigated. 

The visual acuity chart developed in this study asks the subject to choose the answer from among two options, which is both its strength and weakness: It is a weakness in that the subject must be tested on the same optotype multiple times. It is also a strength because it allows asking questions to a 3-year-old using simpler expressions, such as “Which one?” rather than “Which one is this the same as?” in the case of the Lea symbols or HOTV tests. Choosing one of two alternatives may be easier for a child than choosing one from a plurality of three or more. This should, therefore, be considered as an advantage of the novel optotypes. It may be helpful to use both-positive or both-negative optotypes to reduce the probability of justification by chance due to a choice between the two. The optotypes developed in this study also have various pictures drawn around the breaks. Infants and patients with amblyopia have better visual acuity with a single isolated optotype than with many optotypes drawn simultaneously, a phenomenon known as the crowding phenomenon [54]. Therefore, the possibility that the crowding phenomenon may be induced by the optotype developed in this study cannot be ruled out, and further investigation is needed. In addition, young children as young as 3–4 years old are characterized by a preference for color over shape [55]. Red and yellow are particularly preferred colors, followed by blue, green, pink, and orange [56]. It is unclear how visual acuity changes with optotypes such as red and yellow compared to the usual black optotype, and this needs to be investigated in the future, but it is possible that coloring the optotype may be effective in attracting the child’s interest more strongly. These should also be considered in the future. Furthermore, although the optotypes used in this study were familiar to the children, it is expected that familiar optotypes will vary greatly from country to country and ethnic group to ethnic group. In this study, for example, onigiri (rice ball) optotypes were used because the study was conducted in Japan, but these may not be familiar to children in other countries. Therefore, it would be more useful to create optotypes appropriate for each country and ethnic group, but this should be relatively easy because the designs devised in this study are highly versatile.

## 5. Conclusions

In visual acuity testing using the minimum separation threshold, it was shown that by standardizing the width and length of the optotype, it is possible to measure equivalent visual acuity in a variety of designs. In addition, by using the versatile minimum separation threshold, we succeeded in creating a new pediatric acuity chart that can evaluate a minimum separation threshold that did not exist before. Since this study was conducted on a small sample of adults, future studies should be conducted on children to determine the accuracy and success rate of visual acuity measurements using the visual acuity chart developed in this study.

## Figures and Tables

**Table 1:** *p*-value among the three groups.

	*p*-Value
Landolt ring vs. Circle	0.92
Landolt ring vs. Square	0.31
Circle vs. Square	0.40

**Table 2:** Landolt ring, Circle, and Square medians and quartile ranges.

	Q1	Q2	Q3	Interquartile Range
Landolt ring	0.34	0.64	0.72	0.38
Circle	0.34	0.62	0.78	0.44
Square	0.30	0.60	0.76	0.46

## Data Availability

The data presented in this study are available on request from the corresponding author. The data are not publicly available due to privacy protection.

## Supplementary Materials

The following supporting information can be downloaded at: https://www.mdpi.com/article/10.3390/children11040397/s1, LogMAR 1.0 optotype for Landort ring, Circle, and Square.
