# Supplementary figures and images for: Developing a Novel Pediatric Eye Chart Assessing Visual Acuity by Minimum Separable Threshold
Source: Children (Basel). 2024 Mar 27;11(4):397. doi: 10.3390/children11040397 (PMC11048868; doi:10.3390/children11040397)

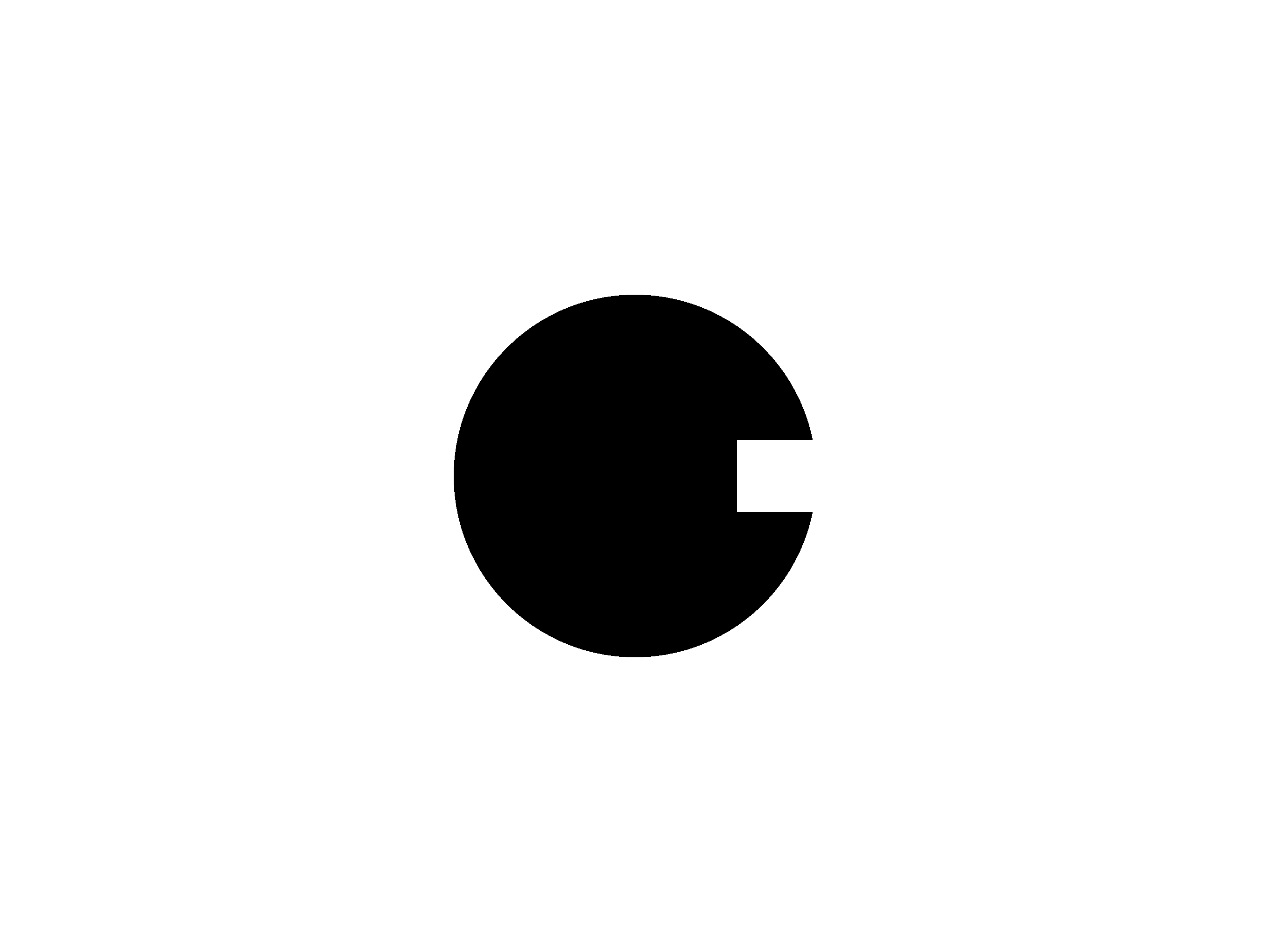

Supplement: Supplementary file 1 [file children-11-00397-s001.zip › Circle_logMAR_1.0.png]

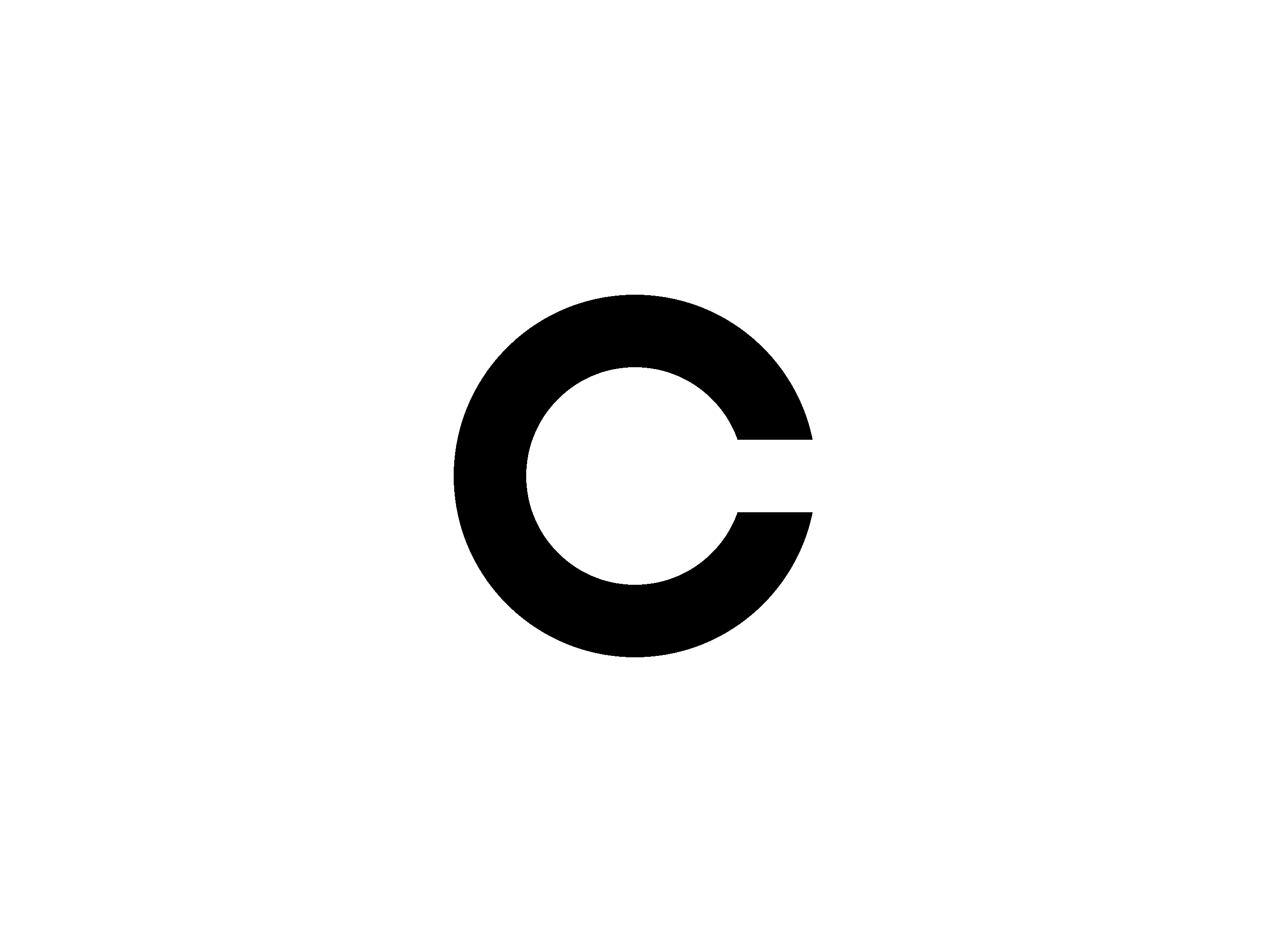

Supplement: Supplementary file 1 [file children-11-00397-s001.zip › Landort_ring_logMAR_1.0.png]

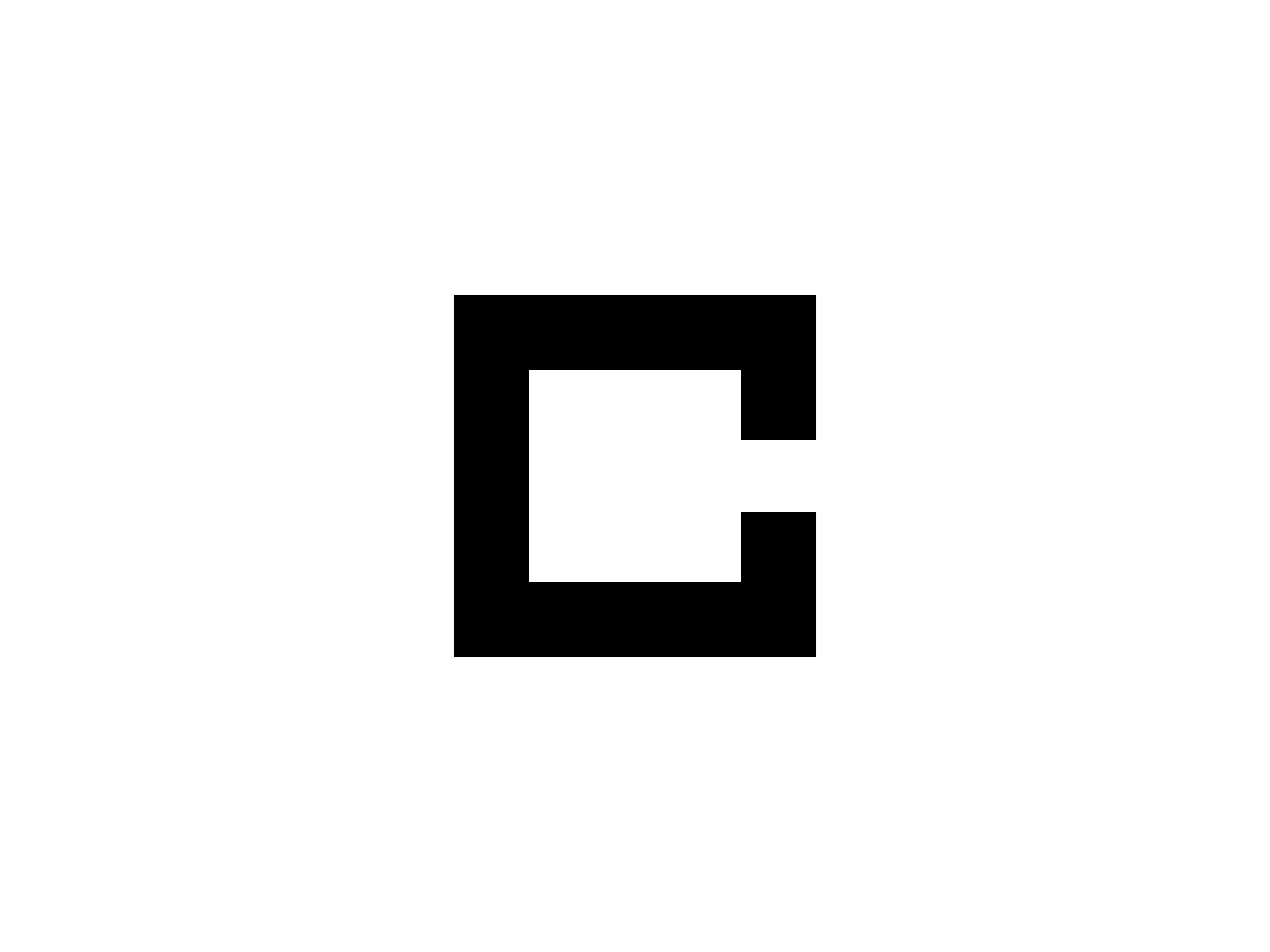

Supplement: Supplementary file 1 [file children-11-00397-s001.zip › Square_logMAR_1.0.png]
